# ASO Author Reflections: Population-Derived Outcome by Treatment-Intent for LAPC

**DOI:** 10.1245/s10434-024-16380-9

**Published:** 2024-10-25

**Authors:** Patrik Larsson, Oskar Swartling, Diana Cheraghi, Ajnon Khawaja, Kjetil Søreide, Ernesto Sparrelid, Poya Ghorbani

**Affiliations:** 1 Division of Surgery and Oncology, Clinical Science, Intervention and Technology, Karolinska Institute, Stockholm, Sweden; 2 Karolinska University Hospital, Huddinge, Sweden

## Past

Several studies report survival for cohorts of patients with locally advanced pancreatic cancer (LAPC) who have undergone resection after neoadjuvant treatment.^1–3^ Favourable outcome may be achieved in the highly selected patients who endure and have stable disease after extensive pre-operative chemotherapy.^4^ However, the outcome for the all-comers of patients with LAPC regardless of the initial treatment recommendation is not well studied. Knowledge of outcomes derived from the entire group of patients presenting with LAPC from time of diagnosis in relation to treatment decision may be valuable for comparison and for patient counselling.

## Present

The current study investigated the survival for all patients with LAPC from the Stockholm region.^5^ Taking advantage of the population-based universal health care system in Sweden, the cohort was unselected without a referral bias. In the cohort, when analysed as an intention-to-treat from diagnosis, patients recommended neoadjuvant chemotherapy did not have a longer survival from time of diagnosis than those recommended chemotherapy with palliative intent. The patients receiving neoadjuvant chemotherapy and undergoing resection had better survival than the patients not undergoing resection. The findings suggest that conversion chemotherapy is successful in only a few select patients, when viewed from a purely population-derived perspective.

## Future

More effective treatment alternatives for patients with LAPC is urgently needed. Predictive markers of therapy failure that may provide an opportunity for therapy switch and better models to identify those patients with LAPC who will benefit from the current treatment alternatives need to be identified. Further studies using an intention-to-treat analysis in a population-based perspective is warranted to understand the real-world outcome for all patients with LAPC.
